# CircTIAM1 overexpression promotes the progression of papillary thyroid cancer by regulating the miR-338-3p/LASP1 axis

**DOI:** 10.32604/or.2024.030945

**Published:** 2024-10-16

**Authors:** YE ZHANG, YANAN LIANG, YAN WU, LIWEN SONG, ZUWANG ZHANG

**Affiliations:** 1 School of Medicine and Health, Jiuzhou Polytechnic, Xuzhou, 221113, China; 2 Department of Oncology, University-Town Hospital of Chongqing Medical University, Chongqing, 401331, China

**Keywords:** Papillary thyroid cancer, Circular RNAs (CircRNAs), LIM/SH3 protein 1 (LASP1), CircTIAM1

## Abstract

**Background:**

Papillary thyroid cancer (PTC) is the most prevalent histological type of differentiated thyroid malignancy. Circular RNAs (circRNAs) have been implicated in the pathogenesis and progression of various cancers. circTIAM1 (hsa_circ_0061406) is a novel circRNA with aberrant expression in PTC. However, its functional roles in PTC progression remain to be investigated.

**Methods:**

The expression levels of circTIAM1 in the PTC and the matched para-cancerous tissues were detected by quantitative real-time reverse-transcription PCR (qRT-PCR). The subcellular localization of circTIAM1 was examined by fluorescence *in-situ* hybridization (FISH). Kaplan-Meier plot was used to analyze the association of clinicopathological features with circTIAM1 expression. Bioinformatics databases were utilized to predict the target miRNAs of circTIAM1 and the downstream target mRNAs. RNA pull-down, RIP assay, and dual-luciferase reporter assay were used to confirm the interactions. Functional experiments, such as CCK-8, EDU staining, and apoptosis assays, as well as *in vivo* xenograft model were employed to explore the impacts of circTIAM1, miR-338-3p, and LIM/SH3 protein 1 (LASP1) on the malignant phenotype of the PTC cells.

**Results:**

CircTIAM1 was highly expressed in PTC cells. Moreover, circTIAM1 silencing suppressed the proliferation and invasion of PTC cells *in vitro* and impaired tumorigenesis *in vivo*. Furthermore, miR-338-3p was verified as a miRNA target of circTIAM1. LASP1 was also identified as a downstream target of miR-338-3p. The anti-tumorigenic effect of miR-338-3p overexpression and the pro-tumorigenic effect of LASP1 was further explored by functional assays, which demonstrated that circTIAM1 modulated the PTC progression through targeting miR-338-3p/LASP1 axis.

**Conclusion:**

The overexpression of circTIAM1 is associated with the malignant progression of PTC. A high level of circTIAM1 promotes the malignancy of PTC cells via the miR-338-3p/LASP1 axis.

## Introduction

Papillary thyroid cancer (PTC) is the major subtype of thyroid cancer which occurs in the endocrine system [1–4]. During the past few decades, the incidence of thyroid cancer has increased substantially in several countries, including USA [1]. According to the World Health Organization statistics, the Western Pacific had the highest incidence of thyroid cancer accounting for 47.6% of total cases worldwide. In addition, the incidence and mortality of thyroid cancer are expected to increase by 29.9% and 67% by 2040, respectively [2]. Despite significant advancement in the therapeutic strategies, such as targeted therapy and immunotherapies, [3] the high incidence of lymph node metastasis and cancer recurrence largely compromise the treatment outcome in patients with PTC [5]. Therefore, deciphering the molecular mechanisms, which contribute to the progression of PTC can undoubtedly facilitate the development of novel therapies for PTC.

Recently, a growing body of evidence indicates that endogenous non-coding ribose nucleic acids (RNAs) and circular RNAs (circRNAs) are implicated in the occurrence and progression of cancers by acting as molecular sponges for microRNAs (miRs) [6–8]. Moreover, circRNAs could also physically interact with proteins to act as protein sponges [9,10]. The deregulation of circRNAs is associated with the malignant progression of PTC [11]. For example, overexpression of circRNA_102171 facilitates PTC progression by modulating catenin beta interacting protein 1-dependent activation of the β-catenin pathway [12]. High level expression of circRNA_102002 promotes the malignancy of PTC cells by regulating the miR-488-3p/hyaluronan synthase 2 (HAS2) axis [13]. circRNA_104565 enhances the proliferation of PTC cells by sponging miR-134 [14]. Although an increasing number of circRNAs has been identified which are dysregulated during cancer progression, their biological functions remain to be fully elucidated [15,16].

The interaction networks of circRNAs and the downstream miRs have gained intensified research interest in different pathophysiological conditions [17]. miRs can act as oncogenic or tumor-suppressive factors and their expressions become deregulated in PTC tissues [18,19]. Delineating the holistic picture of circRNA-miR network can aid the understanding of the molecular mechanisms of PTC development and provide novel targets for therapeutic intervention.

A previous study reported that circTIAM1 (hsa_circ_0061406) was highly expressed in thyroid cancer [20]. However, the functional role of circTIAM1 and its downstream targets in thyroid cancer progression have not been fully unveiled. Therefore, the present study aimed to explore the role and the underlying mechanism of circTIAM1 in PTC. Consistently, it was reported that circTIAM1 was highly expressed in PTC tumor tissues compared with the corresponding expression noted in normal tissues. Elevated circTIAM1 expression was associated with the dismal prognosis of patients with PTC. The potential miR binding sites in the circTIAM1 sequence were predicted and different molecular assays were conducted to confirm that miR-338-3p acted as the downstream mediator of circTIAM1 in PTC cells. Furthermore, LIM and SH3 protein 1 (LASP1) were identified as the protein targets regulated by the circTIAM1/miR-338-3p axis. Overexpression of circTIAM1 facilitated PTC progression by suppressing miR-338-3p and promoting the pro-tumorigenic activity of LASP1. Overall, the data suggest that the circTIAM1/miR-338-3p/LASP1 axis is a critical regulatory module in PTC tumorigenesis and progression.

## Materials and Methods

### Materials and cell lines

PTC cell lines (KTC-1, BCPAP, and TPC-1) and normal thyroid cells (Nthy-ori 3-1) were purchased from the Chinese Academy of Sciences Cell Bank (Shanghai, China). The identity of all cell lines was confrimed by the provider. All cells were tested for the presence of mycoplasma before use. Dulbecco’s modified eagle medium and fetal bovine serum were obtained from Gibco (Rockville, USA). RIPA lysis buffer and ECL solution were purchased from Beyotime Biotechnology (Beijing, China). miR-338-3p mimics/inhibitors and the corresponding controls [(miR-negative control (NC) and inhibitor-NC] and lentiviral particles containing control scramble short hairpin (sh)-RNA (sh-NC) and shRNA targeting circTIAM1 (sh-circTIAM1) were purchased from GenePharma Company (Shanghai, China). The hsa_circTIAM1-overexpressing pLO-ciR vector and LASP1 vector were produced by Geneseed Biotech (Guangzhou, China). To generate a stable cell line with circTIAM1 silencing, the cells were infected with the corresponding recombinant lentivirus at a MOI (multiplicity of infection) = 5 in the presence of 10 µg/ml polybrene (Sigma, Shanghai, China). The infected cells were selected with 1.0 μg/mL puromycin for two weeks to eliminate the uninfected cells prior to further experiments.

### 3D spheroid culture

Tumor spheroids were generated by inoculating PTC cells at a density of 10^3^/well into U-bottom 96-well plates coated with Matrigel (Corning, CA, USA). The cells were cultivated for 5 days until individual spheroids were observed. The number of spheroids was subsequently quantified under a bright-field microscope (Olympus, Tokyo, Japan).

### Clinical sample collection

PTC tumor tissues and the matched para-cancerous normal tissues were collected from 130 patients with PTC from Feb 2017 to Aug 2021 by surgery at the University-Town Hospital of Chongqing Medical University and all the collected specimens were stored at −80°C. The clinicopathological data of the patients are summarized in Table 1. Each patient had provided the relevant informed consent. To avoid the confounding factors which may affect gene expression, the patients who had undergone chemotherapy or radiotherapy were excluded from our analyses. The use of the clinical samples the approval of the Medical Ethics Committee of University-Town Hospital of Chongqing Medical University (LL-201682).

**Table 1:** Association of circTIAM1/miR-338-3p/LASP1 expression and clinicopathologic features of papillary thyroid cancer

Patients		Low circTIAM1 (n = 65)	High circTIAM1 (n = 65)	*p* value	Low miR-338-3p (n = 65)	High miR-338-3p (n = 65)	*p* value	Low LASP1 (n = 65)	High LASP1 (n = 65)	*p* value
**Age (years)**				0.283			0.474			0.72
	≥55	29	23		28	24		25	27	
	<55	36	42		37	41		40	38	
**Gender**				0.415			0.222			0.684
	Male	14	18		13	19		15	17	
	Female	51	47		52	46		50	48	
**Tumor size**				0.157			0.479			0.077
	≥1 cm	24	32		30	26		33	23	
	<1 cm	41	33		35	39		32	42	
**Differentiation**				0.115			0.753			0.753
	Yes	62	57		59	60		59	60	
	No	3	8		6	5		6	5	
**Lymph node metastasis**				0.007			0.212			0.02
	Positive	19	34		30	23		20	33	
	Negative	46	31		35	42		45	32	
**TNM stage**				0.021			0.247			0.007
	I + II	52	40		43	49		53	39	
	III +IV	13	25		22	16		12	26	

### RNA extraction and reverse transcription-quantitative PCR (RT-qPCR)

Total RNA samples were isolated using Qiagen All Prep RNA/Protein mini kit (Sigma, Darmstadt, Germany). The ribosomal RNAs were removed following total RNA extraction. cDNA synthesis was performed using a cDNA synthesis kit (Thermo Fisher Scientific Inc., Waltham, USA). The SYBR Green Master Mix Kit was used to perform quantitative PCR analysis on an ABI 7500 qPCR system (Applied Biosystems, Carlsbad, USA). The 2^−ΔΔCT^ method was used to quantify the relative gene expression, with U6 snRNA as the internal reference for miR and GAPDH as the reference for protein-coding genes and circRNA [11–13]. The GAPDH gene is a highly conserved metabolic gene abundantly expressed in nearly all types of cells. It was used for normalizing LASP1 and circTIAM1 expression levels; U6 encodes an abundant non-coding small nuclear RNA (snRNA) to normalize miR expression. The qPCR primers (Sangon Biotech, Shanghai, China) are summarized in Table A1. The nuclear and cytoplasmic fractioning experiments were performed following extraction of the nuclear and cytoplasmic fractions using NE-PER™ Nuclear and Cytoplasmic Extraction Reagents (Thermo Fisher Scientific, CA, USA). Total RNA in each fraction was subjected to RNA isolation and RT-qPCR analysis.

### Bioinformatic analysis

The sponged target miRs of circTIAM1 were predicted by the circular RNA Interactome online database (https://circinteractome.nia.nih.gov/) [20]. The potential miR binding sites were predicted using “hsa_circ_0061406” as the query. Subsequently, starBase v2.0 software (https://starbase.sysu.edu.cn/starbase2) was employed to predict the downstream targets of miR-338-3p [21].

### Cell transfection

For transfection, the cells were seeded at a density of 5 × 10^4^/well in the 24-well plate and allowed to reach 80% confluence. Subsequently, all oligonucleotides and vectors (Table A2) were transfected to the PTC cell lines using LipofectamineTM 3000 transfection reagent (Invitrogen, Shanghai, China) based on the supplier’s instructions. Following 48 h of incubation, the transfection efficiencies of different groups were examined by RT-qPCR, respectively.

### Cell proliferation and apoptosis assays

The proliferative ability of PTC cells with different treatments was measured by the cell counting kit (CCK)-8 assay. Initially, the transfected cells were cultured at the density of 5 × 10^3^ cells per well in a 96-well plate for different durations. At the indicated time point, the cells were incubated with the CCK-8 reagent (1:100) (Beyotime, Shanghai, China) for 3 h. Subsequently, the optical density values were recorded at 450 nm by using the EnSpire Microplate Reader (Perkin-Elmer, CA, USA). For cell apoptosis detection, the Annexin V-fluorescein isothiocyanate (FITC) apoptosis detection kit (BD Biosciences, CA, USA) was employed according to the manufacturer’s instructions. The cells were incubated for 48 h after transfection and 1 million cells in the staining buffer were labeled with 5 μL Annexin V-FITC and 5 μL propidium iodide reagent for 20 min. Following washing with the staining buffer, the apoptotic events were detected by a BD FACS II flow cytometer (BD Biosciences, CA, USA).

### EDU incorporation assay

Click-iT™ 5-ethynyl-2’-deoxyuridine (EDU) Cell Proliferation Kit for Imaging and Alexa Fluor™ 555 (Thermo Fisher Scietufic, CA, USA) were used to detect DNA synthesis. Pre-warmed EDU solution was added in an equal volume to the cell culture medium for 2 h for labeling. The medium was discarded and the cells were fixed with 100 µL 3.7% formaldehyde in PBS for 15 min at room temperature, followed by permeabilization in 100 µL 0.5% Triton® X-100 for 15 min. The click chemistry was performed using 1 × Click-iT® reaction cocktail based on the manufacturer’s instructions for 30 min. The staining cocktail was removed and the cells were counter-stained with 500 nM 4’,6-diamidino-2-phenylindole (DAPI) in PBS. The cell images were captured under the Leica AM6000 microscope (Leica, Weztlar, Germany).

### Transwell invasion assay

The cell invasive ability was determined using the transwell cassette (Corning, NY, USA) coated with Matrigel. An 8 µm pore insert was pre-coated with 1% Matrigel and the transfected cells (5 × 10^3^) in serum-free medium were inoculated in the upper compartment. The bottom chamber was filled with the complete medium with 10% serum. Following 48 h of incubation, PTC cells that passed through the filter were fixed with 70% ethanol and subsequently stained with 2.5% crystal violet (Sigma, Darmstadt, Germany). Following careful removal of the cells inside the upper insert, the invading cells on the membrane were visualized by a bright-field microscope (Olympus, Tokyo, Japan) [21].

### Dual-luciferase reporter assay

PmirGLO vector expressing firefly luciferase (Promega, WI, USA) was used for luciferase reporter assay. The sequences corresponding to the wild-type binding sites of circTIAM1/miR-330-3p or LASP1 mRNA 3’untranslated region (UTR)/miR-330-3p were cloned into the PmirGLO vector as the wild-type (WT) reporters (circTIAM1-WT or LASP1-WT). The mutated binding sequences of each pair were inserted into the PmirGLO vector as the mutant (MUT) reporters (circTIAM1-MUT or LASP1-MUT). The cells in the logarithmic growth phase were inoculated into 6-well plates at a density of 2 × 10^5^ cells per well, followed by the co-transfection of circTIAM1-WT/LASP1-WT or circTIAM1-MUT/LASP1-MUT reporter with miR-330-3p mimics or the negative control (miR-NC). Following 48 h of incubation, the red firefly and green Renilla luciferase activities were detected using the Luciferase Reporter Gene Assay System (Perkin-Elmer, CA, USA) on the GloMax® discover microplate reader (Promega, WI, USA).

### RNA immunoprecipitation (RIP) assay

The PTC cell lysates were obtained using an immunoprecipitation (IP) lysis buffer (Beyotime, Beijing, China). 10% of lysate was saved as the input for sample normalization. The remaining cell lysate was mixed with anti-argonaute 2 (Ago2) antibody or mouse IgG conjugated-magnetic beads (MCE, Shanghai, China) at 4°C overnight. Following 4 washes with the IP lysis buffer, the co-immunoprecipitated RNA samples on the magnetic beads were extracted using TRIzol reagent (Invitrogen, Shanghai, China) and the relative enrichment of each target was determined by RT-qPCR analysis.

### RNA pull-down assay

The RNA-pull down assay was performed to confirm the physical association between circRNA and miR. Initially, the circTIAM1 or control probe was treated using Biotin-RNA Labeling Mix (Roche, Basel, Switzerland). The cells were lysed with an IP lysis buffer containing protease and phosphatase inhibitor cocktail and RNase inhibitor (Beyotime, Beijing, China). The collected supernatants were mixed with 200 nM biotinylated circTIAM1 or control probe for 4 h at 4°C. The mixture was further incubated with 100 μL Dynabeads™ MyOne™ Streptavidin T1 (Invitrogen, Shanghai, China) for an additional 1 h at 4°C. Following washing of the beads for four times using the IP lysis buffer, the co-precipitated RNA samples on the beads were extracted using TRIzol reagent and the relative enrichment of each target was determined by RT-qPCR analysis.

### Western blotting

Total protein samples were extracted using the RIPA lysis buffer (Beyotime, Beijing, China) and mixed with protease inhibitor and PMSF (ThermoFisher Scientific, CA, USA) following the manufacturer’s instructions. The supernatant was subsequently collected following 30 min incubation by centrifugation (the incubation time for the tissues was prolonged to 4 h and the tissues were sonicated with a tissue breaker prior to lysis). Following denaturation, the protein samples were subjected to separation in 10% or 12% sodium dodecyl sulfate-polyacrylamide gel electrophoresis (SDS-PAGE) and subsequently transferred onto a polyvinylidene fluoride (PVDF) membrane (0.22 or 0.45 µm, Merck-Millipore, Darmstadt, Germany). 5% skimmed milk (w/v) in TBST (Tris-HCl, NaCl, and Tween-20) buffer was used to block the membrane for 2 h. The membrane was subsequently incubated with primary antibodies (Abcam, Cambridge, UK): anti-LASP1 (Abcam, ab156872, 1:1,000) and anti-beta actin antibody [mAbcam 8226] (Abcam, 1:1,000) at 4°C overnight. Subsequently, the PVDF membrane was washed with 1 × TBST buffer and further labeled with horseradish peroxidase (HRP)-conjugated secondary antibody at room temperature [22] for 1 h (Abcam, ab205718, 1:2,000). Following 3 washes, the protein bands were developed using the ECL chemiluminescent solution (Beyotime, Beijing, China).

### In vivo investigation

Balb/c nude mice (male, 4–5 weeks old) were obtained from the Shanghai Laboratory Animal Center Co., Ltd., (Shanghai, China) and housed at the SPF facility. All animal procedures were performed according to the guidelines approved by the Institutional Animal Care and Use Committee of University-Town Hospital of Chongqing Medical University. Initially, the male BALB/c nude mice were divided randomly into two groups (sh-NC or sh-circ-TIAM1, *n* = 5 in each group). Each mouse was inoculated with 5 × 10^6^ KTC-1 cells transfected with sh-NC or sh-circTIAM1. The subcutaneous tumor size was measured every 7 days during 5 weeks with the following the formula: Volume (V, mm^3^) = long diameter × short diameter^2^/2. The mice were euthanized on day 35 and the tumor samples were collected for further analysis. Euthanasia was performed by asphyxiation using a carbon dioxide chamber (flow rate: 35%–45% displacement of the chamber volume per min) for 10 min, which was followed by cervical dislocation. Animal death was confirmed by lack of movement and the cessation of heartbeat and breath. The H&E and the immunohistochemical (IHC) staining analyses were conducted as previously described [11,12].

### Statistical analysis

The data were analyzed by GraphPad Prism software (GraphPad Software, NY. USA) and expressed as mean ± standard deviation. The analysis between the different groups was based on the Student’s *t*-test, one-way or two-way analysis of variance at a defined statistical threshold of *p* < 0.05. The association between circTIAM1 expression levels and the clinicopathological data of patients with PTC was analyzed with the Chi-square test. All the quantitative data are the summary of 3 independent experiments or 3 independent measurements. *, **, and *** denote *p* < 0.05, *p* < 0.01, and *p* < 0.001, respectively.

## Results

### CircTIAM1 is highly expressed in PTC tissues and cell lines

**Figure 1:**
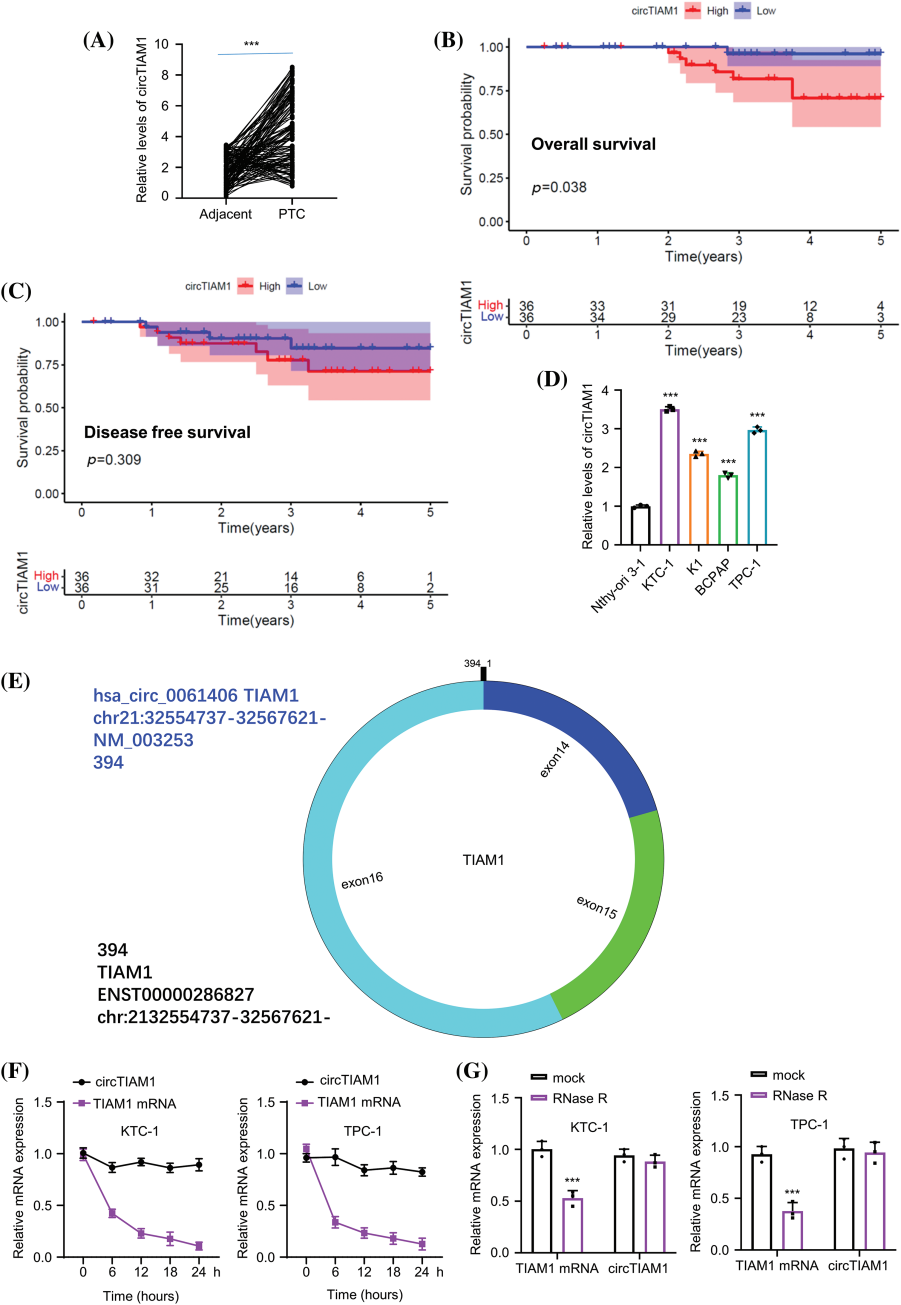
CircTIAM1 is highly expressed in thyroid cancer tissues and cells. (A) qRT-PCR results showing the expression level of circTIAM1 in PTC tumor tissues and matched adjacent normal tissues (n = 130). KM plotter analysis exploring (B) the overall survival of patients with high and low circTIAM1 expression and (C) the disease-free survival curve of patients with different circTIAM1 expressions. (D) The levels of circTIAM1 in thyroid cancer cells and normal thyroid cells (Nthy-ori 3-1 cells). (E) Schematic diagram of circTIAM1 structure: formed by the cyclization of exons 14, 15, and 16 of pre-TIAM1 RNA, with a length of 394 nt (nucleotide). (F) The levels of circTIAM1 and linear TIAM1 mRNA at different time points (0, 6, 12, 18 and 24 h) after actinomycin D treatment in KTC-1 and TPC-1 cells. (G) qRT-PCR showing the expression level of circTIAM1 and TIAM1 mRNA in both cells with or without RNase R treatment. n = 130 clinical samples in (A–C); n = 3 independent experiments in (D, F and G). ***p < 0.001.

To explore the expression pattern of circTIAM1 in PTC, RT-qPCR was performed to detect the levels of circTIAM1 in PTC tissues and in the matched para-cancerous specimens. A significant upregulation of circTIAM1 expression was noted in the PTC clinical samples compared with the para-cancerous specimens (*n* = 130 pairs) (Fig. 1A). Patients with PTC were divided into circTIAM1 low-expression group (*n* = 65) and high-expression group (*n* = 65) based on the median circTIAM1 expression levels (Fig. 1A). The association between circTIAM1 expression levels and the survival of patients with PTC was analyzed (Figs. 1B, 1C). Notably, the overall survival of patients with high levels of circTIAM1 expression was lower than that of the circTIAM1 low-expression group. However, no significant difference was noted in the disease-free survival between the two groups. Owing to the presence of various cell types in cancer tissues, the qPCR results represent the circTIAM1 expression from a mixed cell population. To further validate the overexpression of circTIAM1 in PTC cells, its expression levels were compared between PTC cell lines (KTC-1, BCPAP, and TPC-1) and normal thyroid cells (Nthy-ori 3-1). The results indicated that the expression levels of circTIAM1 were significantly higher in the PTC cell lines (Fig. 1D). The structure of circTIAM1 indicated a cyclization of exons 14, 15, and 16 of pre-TIAM1 mRNA, with a length of 394 nucleotides (Fig. 1E). Since circTIAM1 indicated the highest expression levels in KTC-1 and TPC-1 cell lines (Fig. 1D), these two cell lines were selected and the cells were treated with actinomycin D and RNase R to investigate the stability of circTIAM1. As depicted in Fig. 1F, following blocking of *de novo* transcription by actinomycin D, TIAM1 mRNA levels were dramatically decreased, while cicrTIAM1 levels remained stable for 24 h. Furthermore, RNase R treatment significantly reduced TIAM1 mRNA levels, while no significant change was noted in the circTIAM1 expression following RNase R treatment (Fig. 1G). As shown in Table 1, high circTIAM1 expression levels correlated significantly with the TNM stage and the lymph node metastasis in patients with PTC, while no significant associations were noted between circTIAM1 levels and the following parameters: Patient’s age, sex, tumor size, and differentiation state.

### Knockdown of circTIAM1 expression suppresses the proliferation, invasion, and tumorigenic property of PTC cells

**Figure 2:**
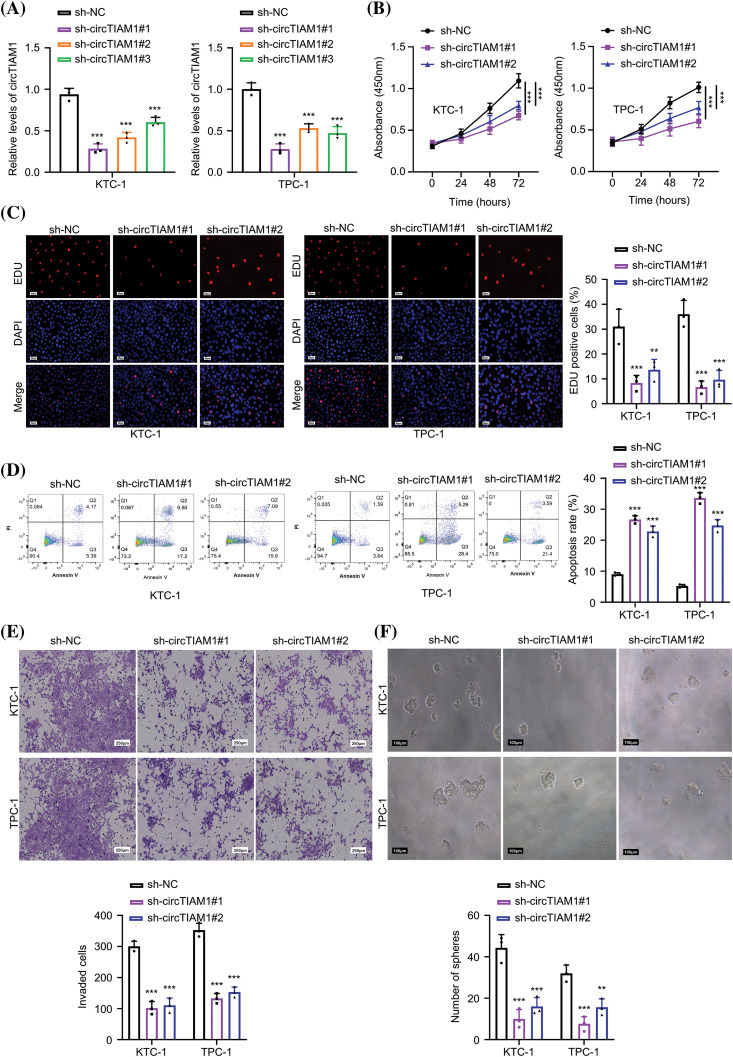
The knockdown of circTIAM1 suppresses cell proliferation, invasion, and tumor spheroid formation of PTC cells. (A) qRT-PCR analysis showing the knockdown efficiency of 3 different circTIAM1 shRNAs in two PTC cell lines (KTC-1 and TPC-1). Cells were stably transduced with lentivirus carrying sh-NC (scramble control sh-RNA), or sh-circTIAM1 #1, #2, and #3 (3 different sh-RNAs targeting circTIAM1). (B) The cell proliferation assay at 0, 24, 48, and 72 h in different groups (sh-NC, sh-circTIAM1#1 and #2). (C) EDU staining of both cell lines in different treatment groups (sh-NC, sh-circTIAM1#1 and #2) (scale bar: 50 μm). (D) The apoptosis levels of PTC cells in different treatment groups (sh-NC, sh-circTIAM1#1 and #2). (E) The transwell invasion assay of cells in different treatment groups (sh-NC, sh-circTIAM1#1 and #2) (scale bar: 1000 μm). (F) 3D spheroid assay of cancer stem-like properties of PTC cells in different groups (sh-NC, sh-circTIAM1#1 and #2) (scale bar: 100 μm). n = 3 independent experiments. **p < 0.01, and ***p < 0.001.

Subsequent experiments were performed to explore the functional roles of circTIAM1 in PTC cells. To this end, KTC-1 and TPC-1 cells were selected with the highest circTIAM1 expression to conduct loss-of-function experiments via circTIAM1 silencing. The stable knockdown of circTIAM1 expression was established by lentiviral infection in both cell lines. Compared with sh-NC (cells expressing control sh-RNA), the sh-circTIAM1 #1, #2, and #3 sequences (cells expressing 3 different sh-RNAs targeting circTIAM1) could effectively reduce circTIAM1 levels in both cell lines (Fig. 2A), among which sh-circTIAM1#1 and #2 indicated an improved knockdown efficiency than sh-circTIAM1#3. Therefore, the sh-circTIAM1#1 and #2 sequences were selected for further experiments. As depicted in Fig. 2B, knockdown of circTIAM1 expression by both shRNA sequences repressed the cell proliferative abilities in both KTC-1 and TPC-1 cell lines, which was further confirmed by the EDU incorporation staining results (Fig. 2C). These data suggest that a high level of circTIAM1 expression is indispensable for the sustained proliferation of PTC cells. Furthermore, a significant increase was noted in the number of apoptotic events in both KTC-1 and TPC-1 cell lines following silencing of circTIAM1 expression (Fig. 2D). Transwell invasion experiments demonstrated that silencing of circTIAM1 expression impaired cell invasion in PTC cells (Fig. 2E). Notably, silencing of circTIAM1 expression further suppressed tumor spheroid formation in the 3D culture (Fig. 2F). Taken together, these data indicate that knockdown of circTIAM1 expression suppresses the proliferation, invasion, and tumorigenic property of PTC cells.

### The targeted interaction between circTIAM1 and miR-338-3p

**Figure 3:**
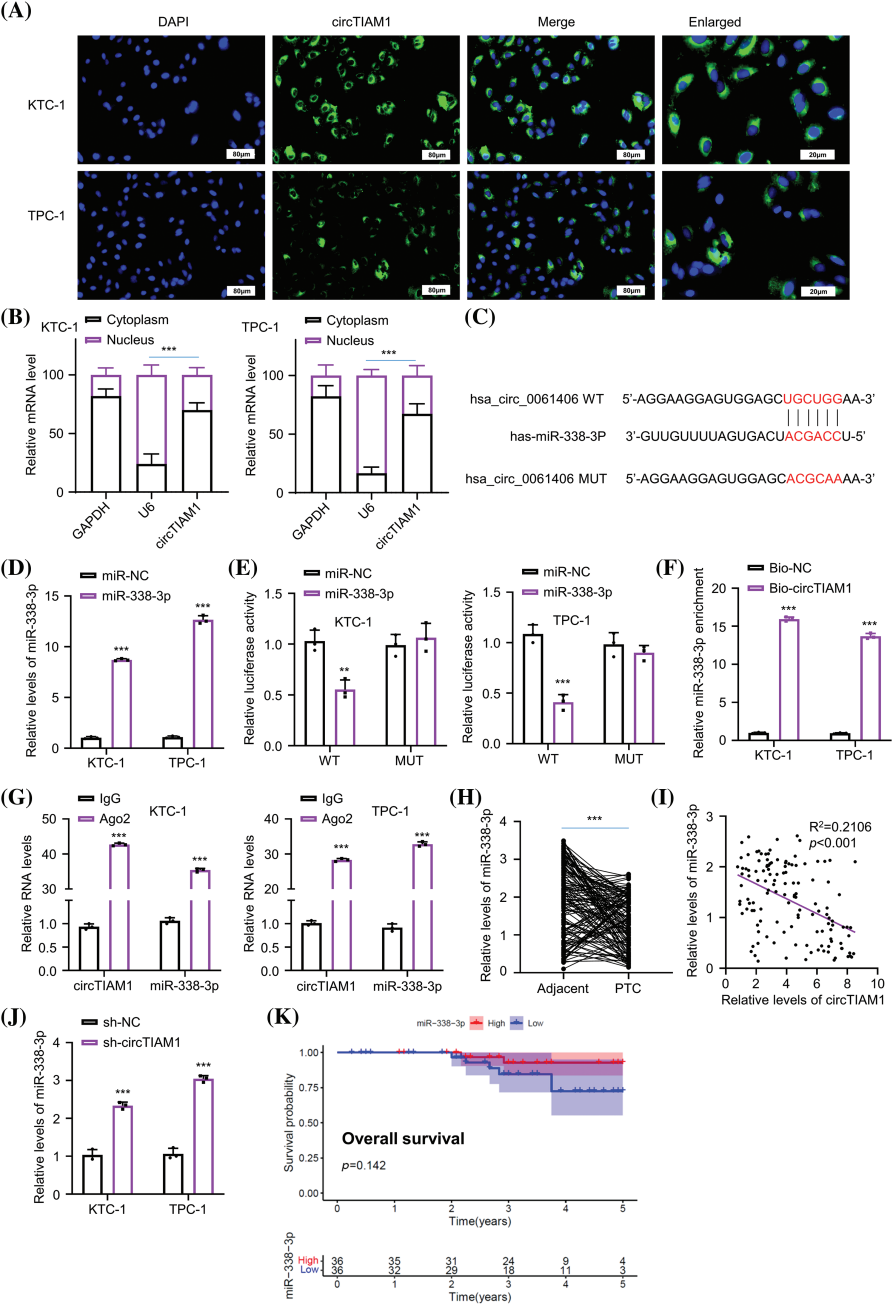
circTIAM1 directly interacts with miR-338-3p. (A) FISH assay showing the cellular localization of circTIAM1 in PTC cells (scale bar: 80 μm). (B) qRT-PCR analysis of the relative abundance of circTIAM1 in the nucleus and cytoplasm compartment. (C) The interaction sequences of circTIAM1 and miR-338-3p as predicted by the circInteractome database. (D) miR-338-3p expression level after the transfection with miR-NC or miR-338-3p mimic. (E) Dual luciferase assay of the circTIAM1 WT or MUT reporter in the presence of miR-NC or miR-338-3p mimic. (F) RNA pull-down assay showing the enrichment of miR-338-3p by circTIAM1 probe. (G) RIP-qRT-PCR results showing the enrichment of circTIAM1 and miR-338-3p by Ago2 antibody. (H) The relative expression level of miR-338-3p in 130 pairs of PTC and adjacent normal tissues. (I) The correlation between the expression of circTIAM1 and miR-338-3p in PTC tumor specimens. (J) The expression level of miR-338-3p after the knockdown of circTIAM1. (K) The overall survival of patients with high and low miR-338-3p expression. n = 130 clinical samples in (H, I and K); n = 3 independent experiments in (B, D–G and J). **p < 0.01, and ***p < 0.001.

**Figure A1:**
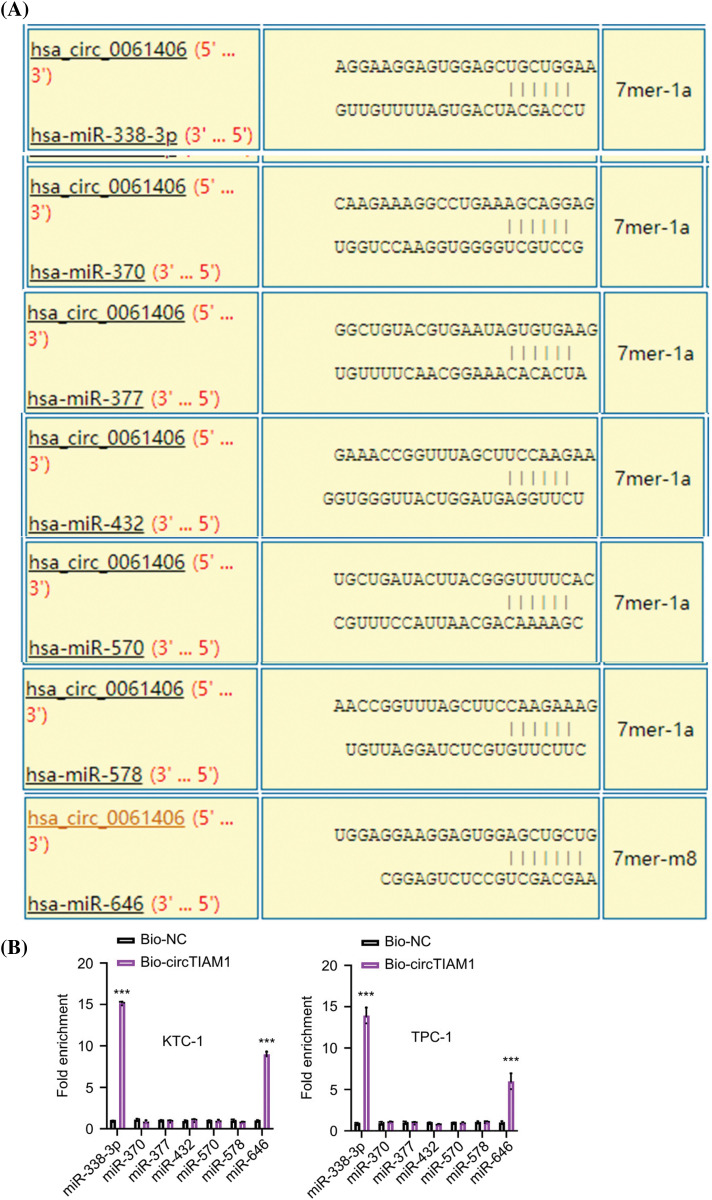
Prediction and analysis of the miRNA candidates of circTIAM1. (A) The miRNA candidates of circTIAM1 predicted by the circInteractome database. (B) RNA pull-down and qRT-PCR analysis of the miRNAs physically associated with biotinylated circTIAM1 probe in KTC-1 and TPC-1 cells. n = 3 independent experiments. ***p < 0.001.

The subcellular localization of circTIAM1 was investigated in PTC cells. As shown by fluorescence *in situ* hybridization staining, circTIAM1 signaling was predominantly observed in the cytoplasm of both KTC-1 and TPC-1 cells (Fig. 3A). The nuclear-cytoplasmic fraction analysis was performed by RT-qPCR. The data demonstrated the relatively higher proportion of circTIAM1 in the cytoplasmic compartment of both cell lines; U6 and GAPDH were used as the internal controls for the nuclear and cytoplasmic fractions, respectively (Fig. 3B). CircRNAs can function as molecular sponges for miRs [5–7]. Therefore, the target miRs of circTIAM1 were predicted using the circInteractome database. Several miRs were predicted as the candidate interacting partners of circTIAM1 (Fig. A1A). An RNA pull-down assay was performed using a biotinylated circTIAM1 probe; the data demonstrated that miR-338-3p and miR-646 were significantly enriched by the circTIAM1 probe in PTC cells, indicating their functional association (Fig. A1B). miR-338-3p was selected as the candidate miR of circTIAM1 for detailed analysis since it was heavily enriched by the circTIAM1 probe. To confirm this functional interaction, the WT and the MUT binding sites between circTIAM1 and miR-338-3p were cloned into the luciferase reporter (Fig. 3C). In addition, miR-338-3p mimic was applied to overexpress miR-338-3p in both cell lines, with miR-NC as the negative control (Fig. 3D). The dual-luciferase reporter assay demonstrated that the co-transfection of miR-338-3p mimic was able to suppress the luciferase activity of the WT reporter. When the predicted binding sites were mutated in the MUT reporter, the inhibitory effect of miR-338-3p mimic was abrogated (Fig. 3E). The RNA pull-down experiment further validated the physical association between the biotinylated circTIAM1 probe and miR-338-3p (Fig. 3F). In addition, RIP-RT-qPCR analysis was performed in both cell lines and the data indicated that the Ago2 antibody could enrich both circTIAM1 and miR-338-3p compared with the IgG control (Fig. 3G), suggesting the functional engagement of circTIAM1 and miR-338-3p in the Ago2-containing RNA processing complex.

Furthermore, the expression levels of miR-338-3p in the PTC and adjacent normal tissues were examined by RT-qPCR (Fig. 3H). It was observed that the expression levels of miR-338-3p were significantly reduced in PTC tissues. The Pearson correlation coefficient was used to analyze the potential association between the expression levels of circTIAM1 and miR-338-3p (Fig. 3I) and the data revealed a trend of negative correlation between circTIAM1 and miR-338-3p. In addition, miR-338-3p expression in PTC cells was increased following knockdown of circTIAM1 expression in both cells (Fig. 3J). Although a tendency of improved overall survival was noted in patients with PTC with high-level miR-338-3p expression, the differences were not significant (Fig. 3K); the expression levels of miR-338-3p were not significantly associated with the clinicopathological features of patients with PTC (Table 1). Overall, these findings imply the negative regulation of miR-338-3p by circTIAM1 in PTC cells.

### miR-338-3p negatively regulates LASP1 protein expression

**Figure 4:**
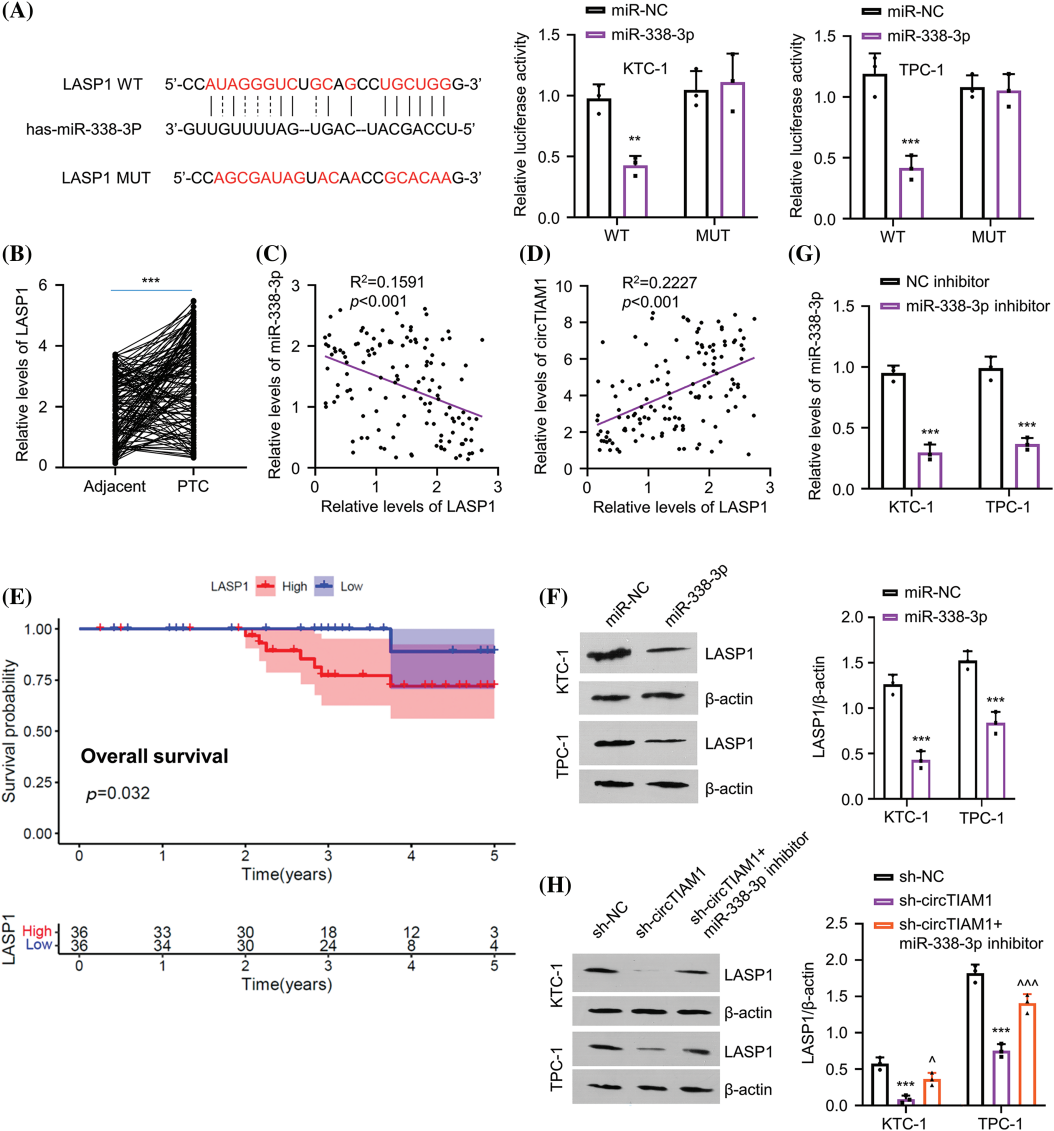
miR-338-3p directly targets LASP1. (A) The StarBase database predicts the binding sites of miR-338-3p at the 3′UTR of LASP1 mRNA, and dual luciferase assay of the LASP1 3′UTR WT or MUT reporter in the presence of miR-NC or miR-338-3p mimic. (B) The relative level of LASP1 in PTC and matched adjacent normal tissues in patients (n = 130). Correlations between (C) LASP1 and miR-338-3p expression, as well as (D) the expression of circTIAM1 and LASP1 in PTC tumor samples. (E) The overall survival of patients with high and low LASP1 expression. (F) The protein level of LASP1 after the transfection of miR-338-3p or miR-NC. (G) The miR-338 expression after the transfection with NC inhibitor or miR-338-3p inhibitor. (H) The level of LASP1 protein in different treatment groups (sh-NC, sh-circTIAM1 and sh-circTIAM1+miR-338-3p inhibitor). n = 130 clinical samples in (B–E); n = 3 independent experiments in (A, F, G and H). **p < 0.01, and ***p < 0.001; ^^^*p* < 0.05, and ^^^^^*p* < 0.001.

**Figure A2:**
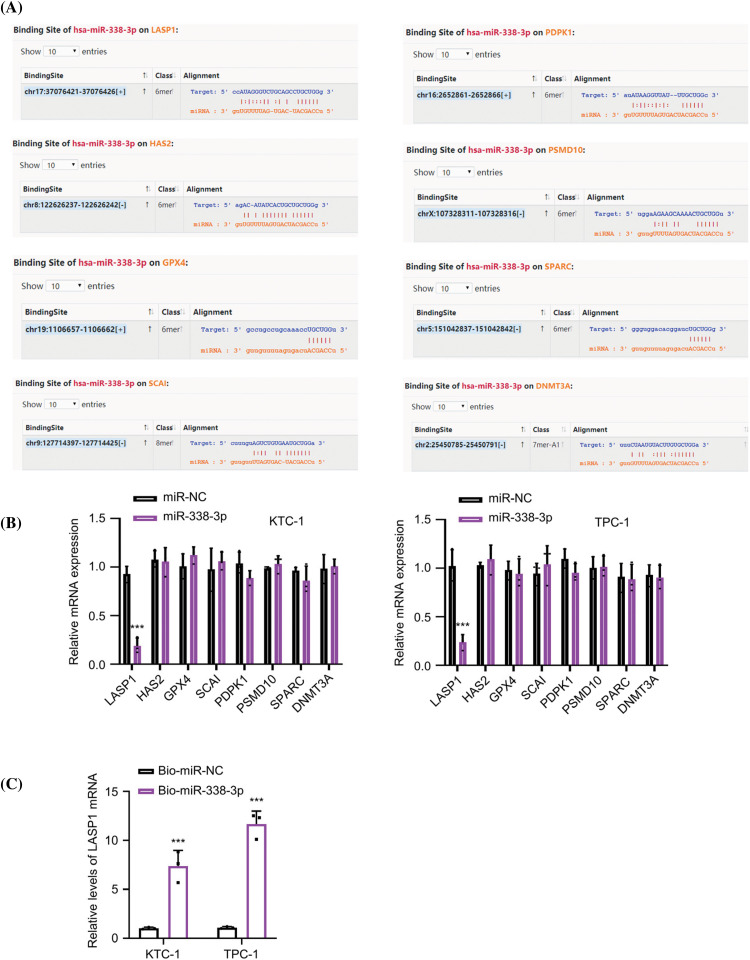
Prediction and analysis of the mRNA candidates of miR-338-3p. (A) The mRNA candidates of miR-338-3p were predicted by the Starbase online resources. (B) The relative expression levels of mRNA candidates after the transfection of miR-NC or miR-338-3p mimic in KTC-1 and TPC-1 cells. (C) RNA pull-down experiment using Biotin-labeled miR-NC or miR-338-3p probe in KTC-1 and TPC-1 cells. n = 3 independent experiments. ***p < 0.001.

The StarBase database was used to explore the downstream mRNA candidates of miR-338-3p, which revealed several mRNAs containing potential miR-338-3p binding sites at the 3′UTR, including the mRNA of LASP1, HAS2, GPX4, SCAI, PDK1, PSMD10, SPARC, and DNMT3A (Fig. A2A). To narrow down the targets, PTC cells were transfected with miR-338-3p mimic and the data indicated that only LASP1 exhibited significant downregulation in its expression levels following miR-338-3p overexpression (Fig. A2B). In addition, RNA pull-down experiments using biotin-labeled miR-NC or miR-338-3p probe indicated that the latter precipitated considerably more LASP1 mRNA in both KTC-1 and TPC-1 cells than the miR-NC probe (Fig. A2C). Their functional interaction was further verified by the dual-luciferase reporter assay using the reporters containing the WT or MUT binding sites (Fig. 4A). A significant upregulation of LASP1 levels was noted in the PTC specimens (Fig. 4B). In addition, a trend of negative correlation was noted between LASP1 and miR-338-3p expressions (Fig. 4C), while the levels of circTIAM1 and LASP1 displayed a trend of positive correlation (Fig. 4D). In patients with PTC, high levels of LASP1 were associated with a lower overall survival (Fig. 4E), and correlated with the TNM stage and lymph node metastasis (Table 1). Following transfection of the cells with miR-338-3p mimic, the protein levels of LASP1 were suppressed (Fig. 4F). A NC inhibitor (the negative control for miR inhibitor) and a miR-338-3p inhibitor (miR antagonist sequence) were also applied in the PTC cells. Compared with the NC inhibitor, the transfection of the cells with the miR-338-3p inhibitor effectively reduced miR-338-3p levels (Fig. 4G). Silencing of circTIAM1 expression in PTC cells reduced LASP1 levels, while co-transfection of the cells with the miR-338-3p inhibitor restored LASP1 protein levels following knockdown of circTIAM1 expression (Fig. 4H). These data imply that LASP1 is the downstream target modulated by the circTIAM1/miR-338-3p axis.

### CircTIAM1 regulates the malignant progression of PTC cells via the miR-338-3p/LASP1 axis

**Figure 5:**
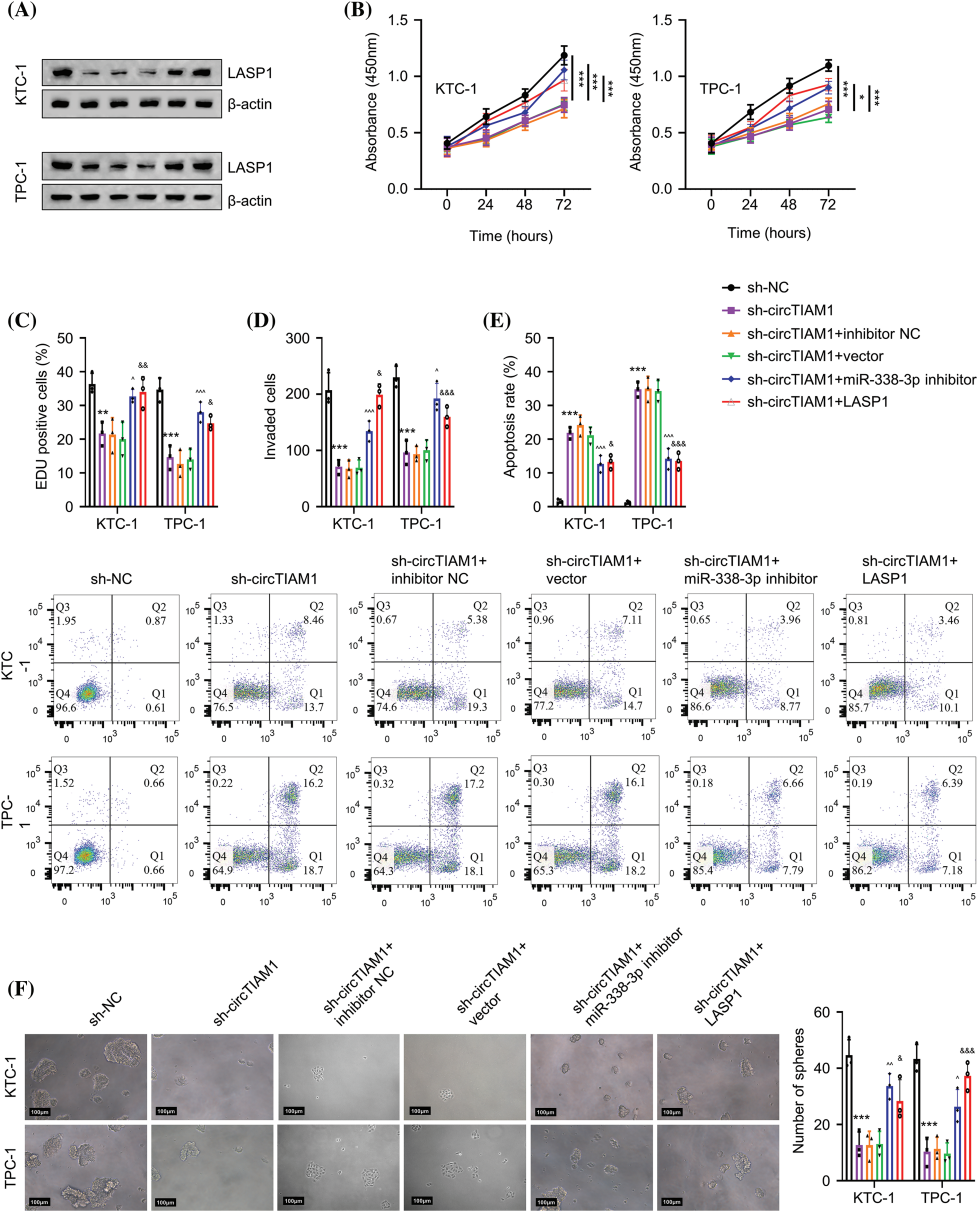
circTIAM1 regulates the malignant progression of PTC cells through miR-338-3p/LASP1. (A) The protein level of LASP1 in cells with different treatment groups (sh-NC, sh-circTIAM1, sh-circTIAM1+inhibitor-NC, sh-circTIAM1+empty vector, sh-circTIAM1 + miR-338-3p inhibitor, and sh-circTIAM1+LASP1). (B) The cell proliferation of cells after different treatments at 0, 24, 48, and 72 h. (C) The EDU incorporation assay in different treatment groups. (D) The transwell invasion assay, (E) The apoptosis analysis by flow cytometry, and (F) 3D spheroid culture of PTC cells in different experimental groups (scale bar: 100 μm). n = 3 independent experiments. *p < 0.05, **p < 0.01, and ***p < 0.001; ^^^*p* < 0.05, ^^^^*p* < 0.01, and ^^^^^*p* < 0.001; ^&^*p* < 0.05, ^&&^*p* < 0.01, and ^&&&^*p* < 0.001.

Subsequent experiments were performed to examine whether circTIAM1 regulates the malignant properties of PTC cells via the miR-338-3p/LASP1 axis. The cells were divided into the following experimental groups: sh-NC, sh-circTIAM1, sh-circTIAM1 + miR-338-3p inhibitor, and the sh-circTIAM1 + LASP1 expression vector. As expected, silencing of circTIAM1 expression reduced LASP1 protein levels, while the transfection of the cells with the miR-338-3p inhibitor and the LASP1 expression vector restored LASP1 protein levels (Fig. 5A). Subsequently, the CCK-8 proliferation assay indicated that the transfection of the cells with the miR-338-3p inhibitor or the LASP1 vector could partially rescue cell proliferation following silencing of circTIAM1 expression (Fig. 5B). Similarly, knocking down circTIAM1 expression reduced the percentage of EDU-positive cells, while inhibition of miR-338-3p expression or LASP1 overexpression increased EDU incorporation in PTC cells, suggesting that inhibition of miR-338-3p expression and LASP1 overexpression promote DNA synthesis and cell growth (Fig. 5C). Inhibition of miR-338-3p expression or LASP1 overexpression could also rescue the cell invasive ability of PTC cells following knockdown of circTIAM1 expression (Fig. 5D). Moreover, the apoptotic cell death induced by silencing of circTIAM1 expression was largely suppressed following transfection of the cells with the miR-338-3p inhibitor and the LASP1 expression vector (Fig. 5E). In the 3D spheroid culture, inhibition of miR-338-3p expression and LASP1 overexpression sustained the tumor spheroid formation following knockdown of circTIAM1 expression (Fig. 5F). Collectively, these results indicate that the miR-338-3p/LASP1 axis mediates the regulatory effect of circTIAM1 in PTC cells.

### Knockdown of circTIAM1 expression inhibits tumor growth in vivo

**Figure 6:**
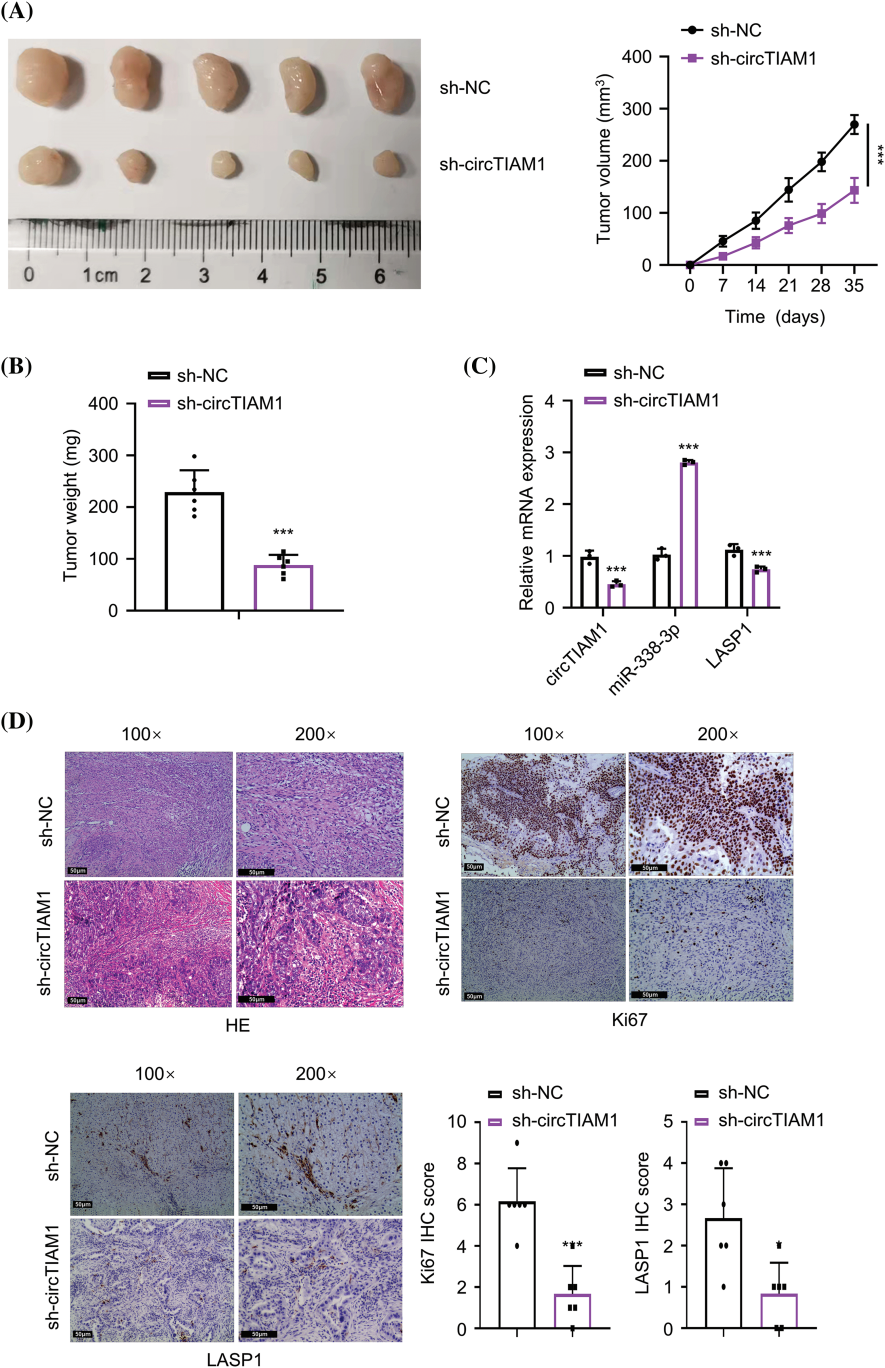
Knockdown of circTIAM1 suppresses the tumorigenesis of PTC cells. (A) The tumor volume curve after the inoculation of KTC-1 cells with sh-NC or sh-circTIAM1 expression. (B) The weight of subcutaneous tumors at the end of the experiment. (C) The levels of circTIAM1, miR-338-3p, and LASP1 in subcutaneous tumors tissues were measured by qRT-PCR. (D) The HE staining, IHC staining of Ki-67 and LASP1 in the subcutaneous tumor sections of the sh-NC and sh-circTIAM1 groups. Data were the summary of IHC staining score of Ki-67 and LASP1 (scale bar: 50 μm). n = 5 mice in each group. *p < 0.05, and ***p < 0.001.

To further evaluate the role of circTIAM1 in the tumorigenesis of PTC cells, KTC-1 cells stably expressing sh-NC or sh-circTIAM1 were grafted subcutaneously in nude mice. The subcutaneous tumor size was measured every 7 days, and the data indicated that knockdown of circTIAM1 expression significantly suppressed tumor growth in nude mice (Figs. 6A and 6B). RT-qPCR analyses in the tumor tissues revealed similar expression patterns of circTIAM1, miR-338-3p and LASP1 in the *in vitro* cell model following silencing of circTIAM1 expression (Fig. 6C). HE staining analysis revealed that the tumor cells were sparsely packed in the xenograft tumor section of the sh-circTIAM1 group. IHC staining of Ki-67 (the cell proliferation marker) and LASP1 indicated the reduced levels of Ki-67 and LASP1 expression in the sh-circTIAM1 group (Fig. 6D). Therefore, silencing of circTIAM1 expression further attenuated tumor cell growth in the animal model.

## Discussion

Over the past decade, non-coding RNAs have become a central topic for exploring the underlying pathological mechanisms of various human diseases [23,24]. With the advancement of the sequencing technologies, the identification of deregulated circRNAs and the investigation of their biological functions have attracted increasing research interest in the field of cancer research. Their structural stability has made circRNAs potential diagnostic biomarkers in different cancer types. In the present study, the overexpression of circTIAM1 was reported in PTC tissues. Moreover, the results indicated an association between high levels of circTIAM1 expression and low survival in patients with PTC. Further analyses demonstrated that circTIAM1 functions as a molecular sponge to dictate the malignant phenotype of PTC cells by negatively regulating miR-338-3p expression. These findings suggest that circTIAM1 regulates the malignant progression of PTC cells by targeting miR-338-3p.

The ability of tumorigenesis is closely related to the tumor spheroid formation in the 3D culture; the inhibition of tumor spheroid growth represents an anti-tumor effect [25]. In the present study, silencing of circTIAM1 expression impaired tumor spheroid formation of PTC cells. Furthermore, knockdown of circTIAM1 expression suppressed the proliferation and invasion of PTC cells. The *in vivo* xenograft mouse model provided additional solid evidence supporting the pro-tumorigenic role of circTIAM1. In line with the present findings, Zhang et al. reported that circTIAM1 promotes PTC progression by targeting the miR-646/HNRNPA1 (Heterogeneous Nuclear Ribonucleoprotein A1) signaling pathway [26]. Overall, the data support the notion that circTIAM1 acts as an oncogenic factor in PTC cells.

miRs are endogenous regulators consisting of 19–22 nucleotides. Their deregulation has been widely implicated in cancer progression [27]. CircRNAs can affect the availability of miRs by adsorbing target miRs in different physiological and pathological processes [28,29]. To identify the miR targets modulated by circTIAM1, bioinformatic prediction and functional validation was performed, which pinpointed miR-338-3p as a key regulator downstream of circTIAM1. Previous studies have reported the abnormal expression of miR-338-3p in various types of cancers [30]. For example, miR-338-3p has been identified as a downstream effector of EGFR overexpression in breast cancer [31]. Increased expression of miR-338-3p suppressed the tumorigenesis of PTC-1 cells in the xenograft mouse model [32]. In addition, miR-338-3p was also reported to suppress cell mobility by downregulating phosphatidylinositol-3,4,5-trisphosphate-dependent Rac exchange factor 2 expression [32]. In the present study, the data indicated that circTIAM1 negatively regulated miR-338-3p activity to modulate cell proliferation and invasive ability. Silencing of circTIAM1 expression promoted miR-338-3p expression levels and suppressed the proliferation and invasion of PTC cells, while inhibition of miR-338-3p expression by the miR-338-3p inhibitor attenuated the effect of circTIAM1 silencing. Based on the inhibitory activity of miR-338-3p on the malignant transformation of PTC cells, the data of the present and previous studies support the notion that miR-338-3p probably acts as a tumor-suppressor in multiple types of cancer.

According to the functional analyses of miR-338-3p targets, LASP1 was negatively regulated by miR-338-3p in PTC cells. LASP1, with a domain of LIM and two-binding domains of actin, interacts with the actin cytoskeleton and transmits signals from the cytoplasm to the nucleus [32,33]. Recently, LASP1 was reported to be highly expressed in different tumor tissues to promote cell proliferation and migration by activation of the PI3K/AKT pathway [34,35]. Moreover, LASP1 overexpression contributes to the aggressiveness of cancer cells [36]. In addition, LASP1 also regulates other signal transduction pathways, cell morphology, and cell motility owing to its association with actin [37,38]. The present study provided novel evidence regarding the mechanism of LASP1 overexpression in PTC tumors. As the downstream target of miR-338-3p, LASP1 is upregulated in PTC cells due to circTIAM1 overexpression and the suppression of miR-338-3p activity. Therefore, circTIAM1 and miR-338-3p act as the upstream regulators of LASP1, thereby modulating the malignant phenotype of PTC cells.

Since circTIAM1 is highly expressed in patients with PTC and could predict a dismal prognosis, circTIAM1 overexpression could be employed as a potential prognostic biomarker. The data further suggest that knockdown of circTIAM1 expression can be developed as a clinical intervention to inhibit malignant progression of PTC. In addition, therapeutic agents which can downregulate LASP1 expression or inhibit its functional activity may also be applied to improve PTC treatment.

The current study presents several limitations. Firstly, we only collected limited number of clinical samples (*n* = 65) in the study to analyze circTIAM1 expression levels and patient survival. A larger sample size can provide more convincing conclusions on the role of circTIAM1 in the clinical progression of PTC. Moreover, given the relatively slow progression of PTC, extended follow-up time of the enrolled patients is required to assess the impact of circTIAM1 expression levels on disease progression. In addition, the mechanism underlying circTIAM1 overexpression in PTC warrants further investigation. The pro-tumorigenic activity of LASP1 needs to be validated in an animal model. Since circTIAM1 may also be secreted in exosomes by cancer cells, future studies can focus on the evaluation of exosomal circTIAM1 in early PTC diagnosis.

## Conclusion

In summary, the present study identified circTIAM1 as a critical oncogenic circRNA supporting the tumorigenesis and malignant progression of PTC. The circTIAM1/miR-338-3p/LASP1 axis may be employed as a promising therapeutic target for the therapeutic intervention of PTC.

## Data Availability

The data analyzed in the present study were submitted as supplementary files in the system and will be deposited into public repository upon acceptance with the URL/DOI provided accordingly.

## Abbreviations

AKT: AKT Serine/Threonine Kinase
CircRNAs: Circular RNAs
CTNNBIP1: Catenin beta interacting protein 1
DMEM: Dulbecco’s modified eagle medium
FBS: Fetal bovine serum
HNRNPA1: Heterogeneous Nuclear Ribonucleoprotein A1
LASP1: LIM and SH3 protein 1
miRNAs: micro RNAs
PI3K: Phosphoinositide-3-Kinase
PREX2: Phosphatidylinositol-3,4,5-trisphosphate Dependent Rac Exchange Factor 2
PTC: Papillary thyroid cancer
RIP: RNA immunoprecipitation
RNAs: Ribose nucleic acids

## Appendix A

**Table A1:** The primer sequences used in qRT-PCR

Gene name	Sequence
LINC00667-F	5′-CTGAAATCACAGCAATGCCAGTTT-3′
LINC00667-R	5′-TATAGCTTTGATTTTCTTGCAGTGT-3′
GAPDH-F	5′-TTTGTCGTATTGGGCCCTGGCA-3′
GAPDH-R	5′-TTGTGTCTTGCTGGGCTGGGGT-3′
miR-338-3p-F	5′-GCGGAGCGCTGCTGCCCTTGG-3′
miR-338-3p-R	5′-GTGGTACTCCTGTCGTGCAA-3′
U6-F	5′-CTCGCTGGTTGCAGCACA-3′
U6-R	5′-AACGCTTCACGAATTGT-3′
LASP1-F	5′-TGCGGCAAGATCGTGTATCC-3′
LASP1-R	5′-GCAGTAGGGCTTCTTCTCGTAG-3′

**Table A2:** The oligonucleotide sequences used for transfection

Gene name	Sequence
hsa_circTIAM1_WT	5′-AGGAAGGAGUGGAGCUGCUGGAA-3
hsa_circTIAM1_MUT	5′-AGGAAGGAGUGGAGCACGCAAAA-3
hsa-miR-338-3P	3′-GUUGUUUUAG--UGAC--UACGACCU-5
LASP1 WT	5′-CCAUAGGGUCUGCAGCCUGCUGGG-3
LASP1 MUT	5′-CCAGCGAUAGUACAACCGCACAAG-3
sh-circTIAM1 #1	5′-GAGATTCTTGAGATCAATAAT-3′
sh-circTIAM1 #2	5′-TTCGAAGGCTGTACGTGAATA-3′
sh-circTIAM1 #3	5′-CTCTTCTATGCTCAAAGATTT-3′
sh-NC	5′-ATATAATCGAGATAGTACTTG-3′
